# Production of Bacterial Cellulose in the Medium with Yeasts Pre-Fermented Coconut Water or with Addition of Selected Amino Acids

**DOI:** 10.3390/foods11223627

**Published:** 2022-11-14

**Authors:** Xue Lin, Zeming Song, Huanyuan Jiang, Yaofei Hao, Xiaoping Hu, Sixin Liu, Congfa Li

**Affiliations:** 1 College of Food Science and Engineering, Hainan University, Haikou 570228, China; 2 College of Sciences, Hainan University, Haikou 570228, China

**Keywords:** coconut water, bacterial cellulose, pre-fermentation, amino acids supplementation, *Komagataeibacter*

## Abstract

The uncontrolled natural pre-fermentation process of coconut water represents great hidden safety hazards, unstable production, and impact on the quality of nata de coco–the trade name of bacterial cellulose (BC) in food industry. In this study, BC production from *Komagataeibacter nataicola* Q2 was conducted in the media of coconut water (50%, *v*/*v*) pre-fermented by 11 coconut-sourced yeast strains in static. Results suggested that coconut water pre-fermented by different yeast strains had varied effects on the production of BC. Compared with the use of fresh coconut water, the use of coconut water pre-fermented by *Saccharomyces cerevisiae* SC7 increased the BC yield by 165%. Both natural pre-fermentation and SC7 pre-fermentation altered the concentrations of amino acids in fresh coconut water. The addition of selected amino acids aspartic acid, glutamic acid, serine, methionine, threonine, isoleucine, phenylalanine, and proline at different concentrations had varied effects on the production of BC. The yield of BC was the highest when adding 3.0% (*w*/*v*) methionine. Moreover, adding 3.0% methionine allowed the production of BC with larger loops of looser aggregated microfibers, increased the crystallinity of BC from 64.8% to 69.4%, but decreased the temperature of maximum weight loss rate, hardness, and adhesiveness from 223 °C, 8.68 kg, and 92.8 g.sec to 212 °C, 7.01 kg, and 58.5 g.sec, respectively, in the test condition.

## 1. Introduction

Nata de coco is the trade name of bacterial cellulose (BC) and has been widely used in beverages, jelly, ice cream, sausage, canning, and baking. Nata de coco has chewy, soft, and smooth texture, contains no cholesterol, low fat, and low calorie, but is rich in fiber, making it a highly popular material for food processing industry worldwide. Nata de coco is fermented by *Komagataeibacter* (previously known as *Gluconacetobacter*, which belongs to *Acetobacter*) spp. mainly using coconut water [1], which is the by-product of coconut processing. Researchers also used other food materials such as citrus peels, pineapple residues, different fruit juices, and thin stillage to achieve cost-effective and enhanced production of BC [2,3].

The production of nata de coco is geographically dependent when using food materials. Using coconut water to produce nata de coco is conducted at the areas where coconut water is produced, such as Hainan of China, Be Tre of Vietnam, and Pontianak of Indonesia. Manufacturers have found that the yield of BC is higher when using coconut water that has been stored for several days prior to fermentation [4,5,6]. Therefore, the natural pre-fermentation method has been widely spread and adopted. However, the natural pre-fermentation process is an uncontrolled process accompanied with the breeding of various microorganisms, resulting in great hidden safety hazards, unstable production, and impact on the quality of nata de coco [1].

Coconut water is rich in nutrients, which is suitable for the growth and reproduction of microorganisms such as yeasts [7]. Yeast cells can utilize the nutrients of coconut water to meet the demand of growth. Simultaneously, the compositions of coconut water are changed by the metabolites of yeast strains. These changes may affect the growth of BC-yielding strains and the synthesis of BC. Literatures have reported that the symbiotic culture of bacteria and yeast (SCOBY), which is commonly used to produce fermented tea beverage Kombucha [8,9,10,11], may be a potential approach for the production of BC [8,9]. Co-culture of BC-yielding strain and yeasts had varied effects on the production of BC. Jia et al. [12] showed that compared with BC-producing strain *Acetobacter xylinum* X-2 monoculture, the production of BC increased by 38.7% with the introduction of *Candida* sp. Y-7. On the contrary, the introduction of a beer yeast of *Saccharomyces cerevisiae* was not conducive to the yield of BC from *Gluconacetobacter hansenii* NOK21 [13]. Compared with the one-step fermentation approach, the pre-fermentation method as a two-step approach may be less efficient in production. However, the competition of nutrition and space might be avoided when using the pre-fermentation method. Several yeast strains were isolated from natural pre-fermented coconut water (NPCW) and stored in our laboratory. Based on this, we made attempts to obtain coconut water pre-fermented by coconut-sourced yeast strains to effectively promote BC synthesis in this study. The artificial-inoculated pre-fermentation strategy is expected to be a substitute for the natural pre-fermentation method to achieve safe, stable, and high-yield production of nata de coco. To the best of our knowledge, few studies research the effect of the yeast pre-fermented medium on the production of BC.

The source of nitrogen, including organic and inorganic nitrogen, is an important factor that affects the growth of microorganisms and the formation of metabolites [14,15]. When organic nitrogen is used, amino acids participate in the regulation of the activity of enzymes involved in the aminoacyl-tRNA synthesis pathway, thereby affecting the synthesis of proteins and a series of catalytic reactions in living cells [16]. The growth of microorganisms and the formation of metabolites could be improved in certain specific amino acids, whereas inappropriate amino acids perform the opposite effects [17,18]. Gomes et al. [18] found that the addition of amino acids had different effects on the production of BC. Three among the 19 amino acids tested, including aspartic acid, phenylalanine, and serine, had more significant positive effects on the synthesis of BC. In addition, the BC membranes displayed lower crystallinity degree but greater thermal and hydrophilic properties in the optimized medium with the supplement of amino acids compared with those produced in a standard condition.

In this study, first, the effect of yeasts pre-fermented coconut water (YPCW) on the production of BC from *Komagataeibacter nataicola* Q2 was investigated, and the yeast strain exerting the most significant promoting effect on BC synthesis was determined. Second, amino acids in pre-fermented coconut water and fresh coconut water (FCW) were comparatively analyzed. Third, the effect of the supplement of selected amino acids (aspartic acid, glutamic acid, serine, methionine, threonine, isoleucine, phenylalanine, and proline) on the synthesis of BC was investigated in the FCW medium. Finally, the characteristics of BC membranes produced from a medium with addition of the key amino acid were analyzed.

## 2. Materials and Methods

### 2.1. Strains and Media

The BC-producing strain *K. nataicola* Q2 and the yeast strains used in this study were stored in the food biotechnology laboratory of Hainan University. The yeast strains included *Candida tropicalis* CT4, CT34, and CT81, *S. cerevisiae* SC7, SC71, SC74, SC84, and SC20, *Issatchenkia orientalis* IO9, *Torulaspora quercuum* TQ134, and *Clavispora lusitaniae* CL117. The relevant taxonomic information about the strains used is shown in Figure S1.

The mature coconuts locally sold in Hainan were broken and the coconut water was filtered to obtain FCW. FCW was placed at room temperature for 3 days to obtain NPCW. A loop of yeast cells was inoculated in 5 mL of yeast extract peptone dextrose (YEPD) medium for culturing at 30 °C for 12 h. The YEPD medium containing 20 g/L of glucose, 20 g/L of peptone, and 10 g/L of yeast extract was sterilized at 115 °C for 20 min before use. Then, 2% (*v*/*v*) cultures were transferred to 40 mL of YEPD medium for culturing at 30 °C for 24 h, from which 2% (*v*/*v*) cultures were transferred to 40 mL of FCW for culturing at 30 °C for 3 days to obtain YPCW. The final content of yeast cells reached approximately 2 × 10^8^ CFU/mL. The *K. nataicola* strain was cultivated in the FCW medium containing 50% (*v*/*v*) FCW, 3.0 g/L of (NH_4_)_2_SO_4_, 0.3 g/L of MgSO_4_, and 0.3 g/L of K_2_HPO_4_. The sugar and pH were adjusted to 8°Brix and 4.5 by sucrose and sodium hydroxide aqueous solution, respectively. BC fermentation was conducted in FCW, NPCW, and YPCW media. The NPCW and YPCW media were prepared similar to the FCW medium with the replacement of FCW by unfiltered and unsterilized NPCW and YPCW, respectively. Therefore, the NPCW and YPCW media contain the cultures of natural microorganism and yeast, respectively. FCW, NPCW, and YPCW media were sterilized at 115 °C for 20 min before use. To investigate the effect of YPCW pre-fermented by the strain SC7 on the production of BC, the SC7 cells were collected from YPCW. After washing with sterile water two times, the SC7 cells were added to the FCW medium, obtaining the SC7-C medium. The fresh coconut water in the FCW medium was replaced by the fermentation supernatants of SC7 in FCW, obtaining the SC7-F medium. YPCW pre-fermented by SC7 was named SC7-PCW for short. The Hestrin and Schramm (HS) liquid medium (pH4.5) contains 20 g/L of glucose, 5 g/L of yeast extract, 5 g/L of peptone, 2.75 g/L of Na_2_HPO_4_, and 1.15 g/L of citric acid.

### 2.2. Fermentation

A loop of *K. nataicola* cells was inoculated in 5 mL of FCW medium at 30 °C for 24 h to obtain the primary pre-culture. Then, 2% (*v*/*v*) cultures were transferred to 40 mL of FCW medium at 30 °C for 24 h to obtain the secondary pre-culture, and 2% (*v*/*v*) cultures (approximately 5 × 10^6^ cells) were inoculated in 40 mL of FCW/NPCW/YPCW/SC7-C/SC7-F media at 30 °C for 5 days in static.

After adding 0.25% (*w*/*v*) or 1.0% (*w*/*v*) aspartic acid, glutamic acid, serine, methionine, threonine, isoleucine, phenylalanine, or proline or 0.5% (*w*/*v*), 2.0% (*w*/*v*), or 3.0% (*w*/*v*) glutamic acid or methionine to the FCW medium, the effect of amino acids on the production of BC was investigated. Fermentations were conducted three times.

After fermentation, BC membranes were filtered and washed using 0.1 mol/L sodium hydroxide solution, 0.5% (*v*/*v*) glacial acetic acid solution, and distilled water in succession. Then, BC membranes were dried to constant weight. The content of BC was expressed as gram (dry weight) of BC membranes per liter of medium.

### 2.3. Determination of Amino Acids

The supernatants of FCW, NPCW, and SC7-PCW were filtered and derivatized using an Agilent automatic on-line derivatization method for the analysis of amino acids according to the previous study [19,20]. The primary and secondary amino acids were reacted with O-phthalaldehyde (OPA) and fluorene methoxycarbonyl chloride (FMOC), respectively.

High-performance liquid chromatography (HPLC) was used to determine the amino acids with an Agilent 1100 apparatus (Agilent, Santa Clara, CA, USA) and a ZORBAX Eclipse AAA column (4.6 mm × 150 mm, 3.5 μm, Agilent, Santa Clara, CA, USA). 40 mM sodium dihydrogen phosphate (pH7.8) was used as the mobile phase A. The mobile phase B contained acetonitrile, methanol, and water (45:45:10, *v*/*v*/*v*). The gradient was 0% B (0 min), 0% B (1 min), 57% B (23 min), 100% B (27 min), 100% B (34 min), 0% B (40 min), and 0% B (41 min). The mixed standard of 17 kinds of amino acids (Sigma, Saint Louis, MO, USA), including aspartic acid, glutamic acid, serine, histidine, glycine, threonine, arginine, alanine, tyrosine, cysteine, valine, methionine, phenylalanine, isoleucine, leucine, lysine, and proline, and the tryptophan standard (Sigma, Saint Louis, MO, USA) were used in the identification and quantification. Experiments were conducted in triplicate.

### 2.4. Determination of Growth

*K. nataicola* cells were inoculated in the FCW medium at 30 °C for two consecutive times of pre-culture. 2% (*v*/*v*) of the secondary pre-culture was inoculated in 40 mL of FCW medium with or without methionine at 30 °C, and the cell density OD600 was monitored using a UV spectrophotometer (UV-5500(PC), METASH, Shanghai, China). Experiments were conducted in triplicate.

### 2.5. Determination of Gluconic Acid Content

The content of gluconic acid was determined by HPLC according to the previous study [1,21]. An Agilent 7890A GC (Agilent, Santa Clara, CA, USA) coupled with an Agilent 1260 chromatograph (Agilent, Santa Clara, CA, USA) was used. The chromatograph was equipped with a DAD detector and a ZORBAX-SB-Aq column (250 mm × 4.6 mm, Agilent, CA, USA) at 30 °C. Perchloric acid aqueous solution (pH2.5)-methanol (98:2, *v*/*v*) was used as mobile phase at a flow rate of 0.6 mL/min.

### 2.6. Analyses of Morphology, Fourier-Transform Infrared (FTIR) Spectrum, X-ray Diffraction (XRD), and Thermogravimetry (TG)

Freeze-dried BC membranes were characterized using micrographs, FTIR spectroscopy, XRD, and TG analyses. The morphological characteristics of BC membranes were observed by a scanning electron microscope (SEM, S-3000N, Hitachi, Tokyo, Japan). FTIR spectra were recorded using a spectrometer (Tensor 27, Bruker, Karlsruhe, Germany) with a resolution of 4 cm^−1^, ranging from 4000 cm^−1^ to 400 cm^−1^. XRD patterns of the BC membranes were obtained using an X-ray diffractometer (SmartLab, Rigaku, Tokyo, Japan) with Cu-Kα radiation. The operating voltage and electric current were 10 kV and 100 mA, respectively. The 2θ scans were made in the range between 5° and 40° at a speed of 5°/min. The degree of crystallinity (crystallinity index, CrI) was determined by the following equation: CrI(%) = (I_002_ − I_AM_)/I_002_ × 100%, where I_002_ represents the maximum intensity of the lattice diffraction at approximately 2θ = 22.6°, and I_AM_ represents the diffraction intensity in the same units at approximately 2θ = 18° [22]. Thermogram analysis, which was used to investigate the thermal stability of the BC membranes [23], was conducted using a thermal analyzer (Q600 SDT TGA, TA, NewCastle, DE, USA) at a heating rate of 10 °C/min. Curves of TG and a derivative form of TG (DTG) were recorded.

### 2.7. Analysis of Texture Characteristics

The texture characteristics of fresh BC membranes were analyzed by textural profile analysis (TPA) using a texture analyzer (TA.XT Plus, SMS, London, UK) equipped with a probe P/36R. The test speed and trigger force in TPA were 1 mm/sec and 5 g, respectively. The purpose of trigger is to let the instrument judge whether there is contact with the sample. The inertia of running at 1 mm/sec is not greater than 5 g. Setting the force to 5 g can ensure that the test only starts when the sample is contacted. The deformation level was 30% of the original height of samples. The measurements were conducted in triplicate.

### 2.8. Statistical Analysis

Results were expressed as average ± standard deviation (SD). Differences of the experimental group and the control group were determined using Student’s *t*-test and differences of *p* < 0.05 were considered as statistically significant.

## 3. Results

### 3.1. Production of BC from K. nataicola in the YPCW Media

**Figure 1:**
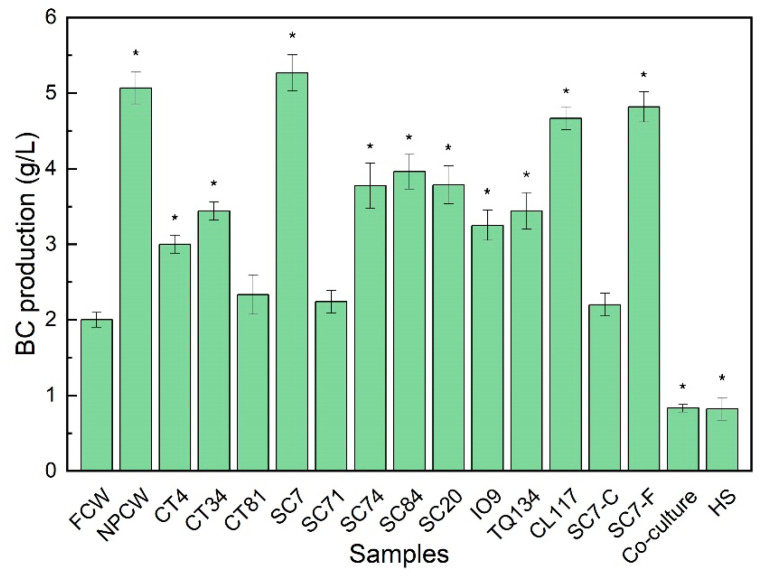
Production of BC in different coconut water media. FCW: fresh coconut water medium; NPCW: natural pre-fermented coconut water medium; CT4, CT34, CT81: the media of coconut water pre-fermented by *Candida tropicalis* CT4, CT34, and CT81, respectively; SC7, SC71, SC74, SC84, and SC20: the media of coconut water pre-fermented by *Saccharomyces cerevisiae* SC7, SC71, SC74, SC84, and SC20, respectively; IO9: the medium of coconut water pre-fermented by *Issatchenkia orientalis* IO9; TQ134: the medium of coconut water pre-fermented by *Torulaspora quercuum* TQ134; CL117: the medium of coconut water pre-fermented by *Clavispora lusitaniae* CL117; SC7-C: the medium of fresh coconut water containing *S. cerevisiae* SC7 cells; SC7-F: the medium of coconut water pre-fermented by *S. cerevisiae* SC7 without cells; Co-culture represents the co-culture of *K. nataicola* Q2 and *S. cerevisiae* SC7 at a ratio of 1:1 (*v*/*v*) in the FCW medium; HS: HS medium. Significant differences of the samples to the control group FCW were confirmed at * *p* < 0.05 (n = 3).

In this work, the production of BC from *K. nataicola* Q2 was conducted using media of FCW, NPCW, and YPCW pre-fermented by 11 yeast strains. The yield of BC in the pre-fermented coconut water media was obvious different from that in the FCW medium (Figure 1). BC production in the media of YPCW pre-fermented by stains CT81 and SC71, respectively, was similar to that in the FCW medium, whereas using YPCW pre-fermented by other 9 stains increased the yield of BC to different degrees. This result demonstrated that the coconut water pre-fermented by different yeast strains had varied effects on the production of BC from *K. nataicola* Q2. The production of BC in the SC7-PCW medium was the highest, which was even slightly higher than that in a NPCW condition. Therefore, using the pre-fermented coconut water by SC7 is expected to be a potential alternative strategy to achieve safety, stable, and high-yield production of nata de coco.

The effect of the co-culture of *K. nataicola* Q2 and *S. cerevisiae* SC7 on the production of BC was also investigated in the FCW medium. Surprisingly, compared with *K. nataicola* Q2 monoculture, the yield of BC produced in the co-culture system of *K. nataicola* Q2 and *S. cerevisiae* SC7 in the tested condition was lower (Figure 1). This finding confirms that the effect of co-culture with yeasts on the production of BC is largely depended on the yeast strains used [12,13].

Yeasts exert an effect on the production of BC in at least two aspects. On one hand, yeast cells contain rich nutrients such as vitamins, amino acids, and nucleotides [24]. On the other hand, yeast strains can metabolize and produce/consume promoters or suppressors to disturb the growth and metabolism of BC-producing strains. To further investigate the effect of YPCW on the production of BC, BC productions from *K. nataicola* Q2 in the media of SC7-C and SC7-F were tested. The production of BC in the SC7-F medium was similar to that in the media of NPCW and SC7-PCW. Nevertheless, a lower BC production was found in the SC7-C medium, which was similar to that in the FCW medium. This result suggested that the metabolites of SC7 might be more important than the yeast cells to the improvement of BC in a pre-fermented condition in this work, though yeast extracts were used to increase BC yield in another study [25]. Literatures have shown that appropriate concentrations of ethanol, acetic acid, or pyruvic acid can promote the growth of BC-yielding strain and the synthesis of BC to varying degrees [26,27,28]. We also found that exogenous addition of ethanol, acetic acid, or pyruvic acid had different effects on the production of BC (Figure S2). In the future study, more pre-fermentation metabolites of the SC7 strain and natural pre-fermentation metabolites related to the change of BC yield can be explored using metabolomics technology.

### 3.2. Amino Acids in FCW, NPCW, and SC7-PCW

**Figure 2:**
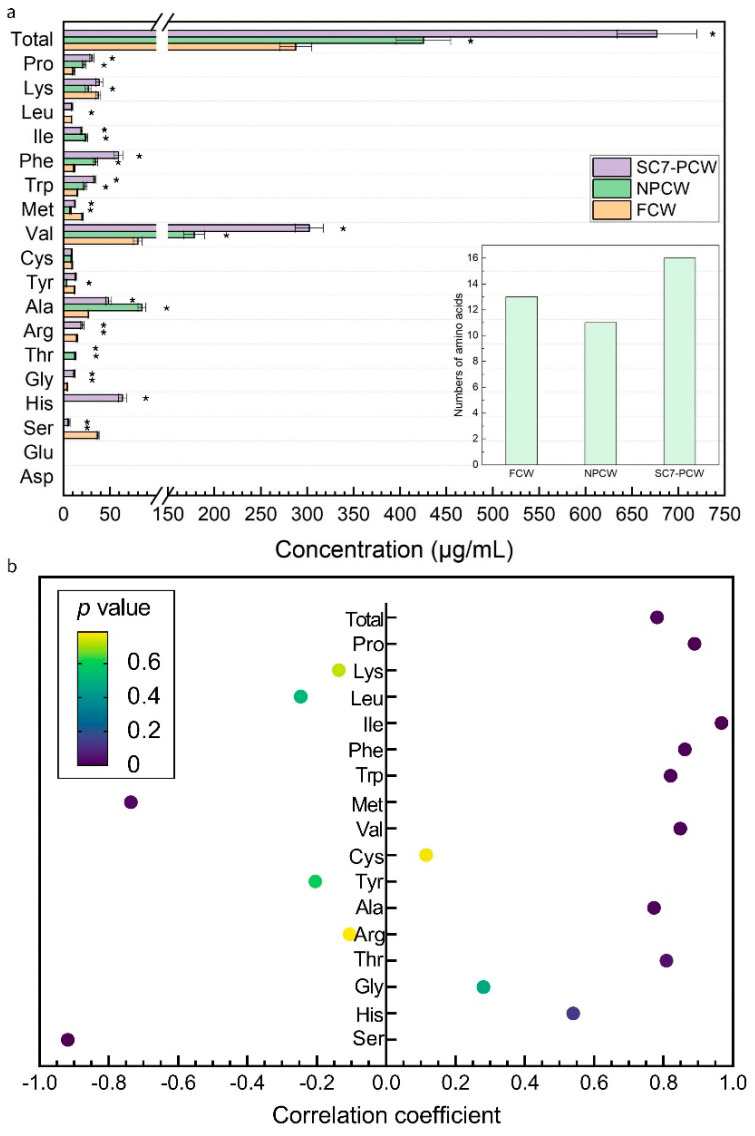
Amino acids in FCW, NPCW, and SC7-PCW (**a**) and Pearson’s correlation coefficient analysis between the contents of amino acids and BC yield (**b**). FCW: fresh coconut water; NPCW: natural pre-fermented coconut water; SC7-PCW: coconut water pre-fermented by *S. cerevisiae* SC7. Significant differences of the samples to the control group FCW were confirmed at * *p* < 0.05 (n = 3).

The composition of coconut water inevitably changed in natural or yeast pre-fermentation. As an important nutrient element, amino acid is not only important for the growth of organisms, but also participates in the biological metabolism via direct and indirect regulatory pathways [29]. Therefore, amino acids in FCW, NPCW, and SC7-PCW were analyzed. Compared with FCW, the variety and concentration of amino acids were different in NPCW and SC7-PCW (Figure 2a). Compared with FCW, the variety of amino acids decreased from 13 to 11 in NPCW, but SC7-PCW increased the variety of amino acids to 16. Notably, the contents of serine and methionine decreased in NPCW and SC7-PCW, but the total content of amino acids increased compared with that in FCW. The highest total content of amino acids was observed in SC7-PCW. Yeast cells could decompose proteins and accumulate amino acids in the early stage of fermentation, and amino acids were utilized in the later stage [30]. NPCW had complex microorganisms [1], which may be unfavorable to the accumulation of amino acids in NPCW. In other words, the increased amino acids in NPCW could not be attributed to SC7 only.

Correlation between the concentration of amino acids and the production of BC was analyzed by Pearson’s correlation coefficient analysis and the following three cases displayed (Figure 2b): (1) the contents of threonine, alanine, valine, tryptophan, phenylalanine, isoleucine, proline, and total content of amino acids were significantly positively correlated with the yield of BC; (2) the contents of serine and methionine were significantly negatively correlated with the yield of BC; (3) no significant correlations were observed between the contents of other seven amino acids (histidine, glycine, arginine, tyrosine, cysteine, leucine, and lysine) and the yield of BC. These results demonstrated that the effect of amino acids on the synthesis of BC might depend on the variety and concentration of amino acids, which was consistent with the influence of amino acids on the synthesis of metabolites of *Candida glycerinogenes* [31] and *S. cerevisiae* [32].

### 3.3. BC Production from K. nataicola with the Addition of Selected Amino Acids

**Figure 3:**
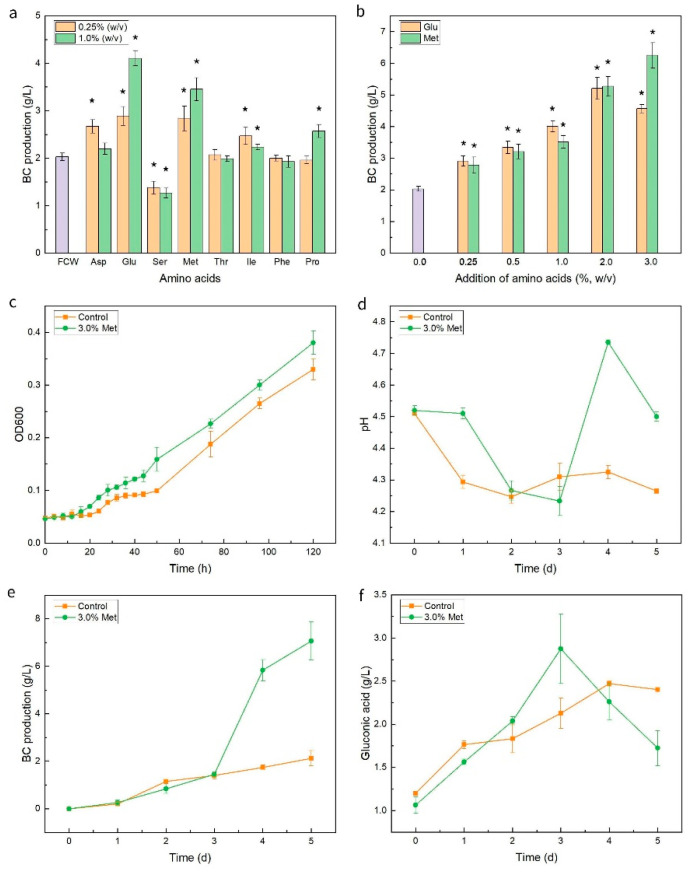
Effects of addition of selected amino acids on the production of BC (**a**,**b**), and addition of 3.0% (*w*/*v*) methionine on the synthesis of BC (**c**–**f**). Control: the BC membrane obtained in the FCW medium; 3.0% Met: the BC membrane obtained in the FCW medium with the addition of 3.0% methionine. Significant differences of the samples to the control group FCW without addition of amino acids were confirmed at * *p* < 0.05 (n = 3).

To verify the effect of amino acids on the production of BC, aspartic acid and glutamic acid (the two that did not present in FWC, NPCW, and SC7-PCW), threonine and isoleucine (the two that only appeared in NPCW and SC7-PCW), serine and methionine (the two that decreased in both NPCW and SC7-PCW compared with FCW), and phenylalanine and proline (the two that increased in NPCW and SC7-PCW compared with FCW), were selected and separately added to the FCW medium. Compared with FCW medium without the addition of amino acids, the production of BC increased when adding 0.25% aspartic acid, glutamic acid, methionine, or isoleucine, but decreased when adding 0.25% or 1.0% serine (Figure 3a). No obvious changes in BC yield were observed when adding 0.25% threonine, phenylalanine, or proline and 1.0% aspartic acid, threonine, or phenylalanine. These results reflected that the exogenous addition of different concentrations of amino acids had varied effects on the yield of BC. Gomes et al. [18] showed that the addition of aspartic acid, phenylalanine, and serine had a significant positive effect on the production of BC in a static culture condition. Son et al. [33] found that the addition of tyrosine, valine, methionine, isoleucine, or glycine decreased the BC yield from *Acetobacter* sp. V6 under shaking culture conditions. Heo et al. [34] showed that single omission of threonine could reduce the formation of BC. Differences of these results could be caused by the differences of the strain used and/or culture conditions.

The yield of BC was relatively high after the addition of glutamic acid or methionine. With the increase in the concentrations of glutamic acid and methionine below 2.0% and 3.0%, respectively, the BC production increased (Figure 3b). Most notably, the content of methionine was significantly negatively correlated with the production of BC (Figure 2b), but the addition of methionine enhanced the BC yield at concentrations above 0.25%. Next, a smaller amount of methionine was added to the FCW medium. Compared with the FCW medium without the addition of methionine, the yield of BC reduced from 1.99 ± 0.15 g/L to 1.60 ± 0.07 g/L and 1.59 ± 0.18 g/L when adding 0.02% and 0.05% methionine, respectively, suggesting the influence of concentration of amino acids in the synthesis of BC.

The highest yield of BC was obtained with the addition of 3.0% methionine, which was 207.9% higher than that in the control group (Figure 3b). S-adenosylmethionine (SAM) participates in the important biochemical reactions in vivo as methyl donors and precursors of physiological thiocompounds [35]. SAM is also essential for the production of polyamines, N-acylhomoserine lactone (autoinducer–1), vitamins, and other biomolecules [35]. The addition of methionine might promote the synthesis of BC via the aforementioned pathways mediated by SAM. Compared with the control group, the growth of *K. nataicola* Q2 was enhanced when adding 3.0% methionine (Figure 3c). This result was consistent with the report that methionine stimulated the growth rate of *A. xylinum* subsp. *sucrofermentans* BPR 2001 at the early stage of culture and increased the production of BC [36]. The production of BC increased with promoted growth of *K. nataicola* Q2, but the content of gluconic acid as a byproduct was lower at the same condition (Figure 3f), suggesting that the addition of methionine may promote the BC flux and inhibit the formation of gluconic acid. The mechanism of methionine and other amino acids in the regulation of BC synthesis needs to be further studied in future.

### 3.4. Characterization of BC Membranes Obtained with the Addition of 3.0% Methionine

**Figure 4:**
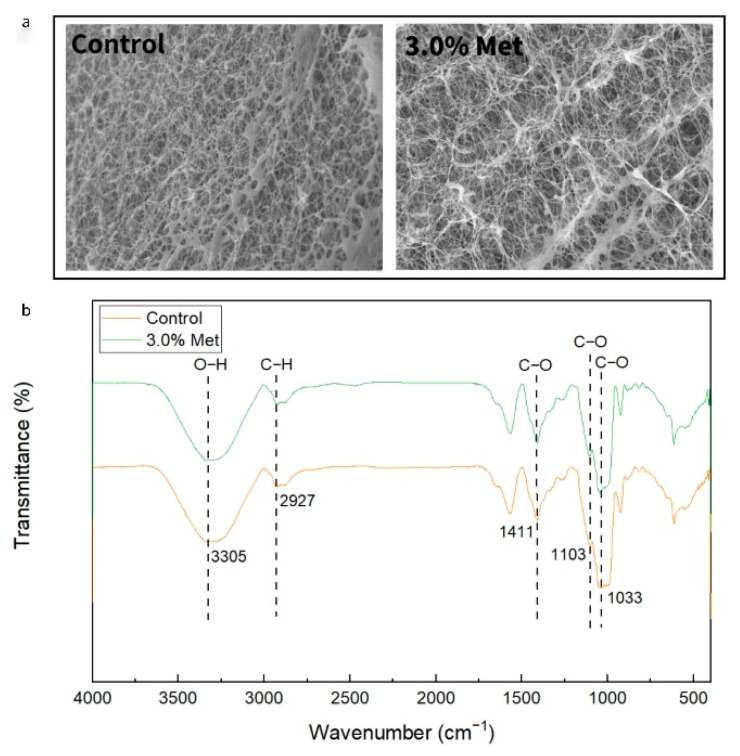
SEM images (**a**) and FTIR spectrogram (**b**) of BC membranes. The working distances of SEM images for the control and 3.0% Met samples were 16.8 mm and 17.2 mm, respectively, under 2.7k× magnification and with a scale of 10 μm. The acceleration voltage was 10.0 kV. Control: the BC membrane obtained in the FCW medium; 3.0% Met: the BC membrane obtained in the FCW medium with the addition of 3.0% methionine.

The cross-section morphology of BC membranes obtained in the FCW media with and without the addition of 3.0% methionine was observed using SEM (Figure 4a). Compared with the control group, the fine microfibers were thinner, longer, and looser, and aggregated to form larger loops under the addition of 3.0% methionine. This result demonstrated that the addition of methionine might affect the extracellular activity of BC synthesis. This finding was consistent to the results of Seto et al. [37], which conjectured that the improved synthesis of BC may be observed in the process of cellulose fibers assembly in a co-culture system of *Gluconacetobacter xylinus* and *Lactobacillus mali*. Such a BC membrane has a great application prospect in not only the textile industry due to the increased comfort and dyeing characteristics of textiles, but also in the development of medical materials, such as artificial skin and wound dressing [38,39,40].

The functional groups of BC membranes obtained with and without the addition of 3.0% methionine were analyzed by FTIR spectroscopy. Compared with the control group, no new peaks were observed under the addition of 3.0% methionine (Figure 4b), suggesting that the addition of 3.0% methionine did not evidently affect the FTIR spectrum of BC. This finding was consistent with the result of a previous study [18], where BC membrane was produced in an optimized medium supplemented with amino acids. The signal bands around 900–1100 cm^−1^ became weaker with the addition of 3.0% methionine, which may be related to the stretching vibration of C–O and C–C from differential metabolites in FCW medium with and without the addition of methionine.

**Figure 5:**
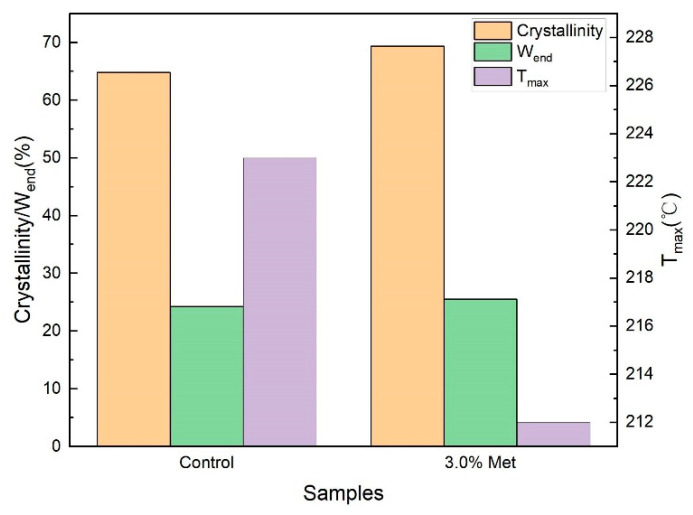
Crystallinity degree, W_end_, and T_max_ of BC membranes. W_end_: the final weight loss rate of thermal degradation; T_max_: the temperature of maximum weight loss rate. Control: the BC membrane obtained in the FCW medium; 3.0% Met: the BC membrane obtained in the FCW medium with the addition of 3.0% methionine.

XRD patterns and TG curves were monitored (Figure S3). The crystallinity degree, the final weight loss rate of thermal degradation (W_end_), and the temperature of maximum weight loss rate (T_max_) are shown in Figure 5. The crystallinity degree of the BC membrane obtained with the addition of 3.0% methionine was 7.0% higher than that in the control group. A high crystallinity was accompanied with a high yield of BC under the addition of 3.0% methionine, which was consistent with the study in a standard HS medium [18]. The mechanical property of BC might be enhanced under high crystallinity, which could increase the strength and durability of paper and is beneficial for the application of papermaking (e.g., the production of special paper) [41]. The W_end_ of BC membrane was 24.25% and 25.53% without and with the addition of 3.0% methionine, respectively; correspondingly, T_max_ was 223 °C and 212 °C, respectively. These results were consistent with the findings of XRD with more stable I_β_ transforming to unstable I_α_, suggesting that the addition of 3.0% methionine slightly reduced the thermal stability of BC from *K. nataicola* Q2. However, this finding was not consistent with the results of the previous study [18], where the supplementation of amino acids (aspartic acid, phenylalanine, and serine) resulted in lowering the crystallinity but increasing the thermal properties. This discrepancy might be resulted from the differences of variety and concentration of supplemented amino acids.

**Figure 6:**
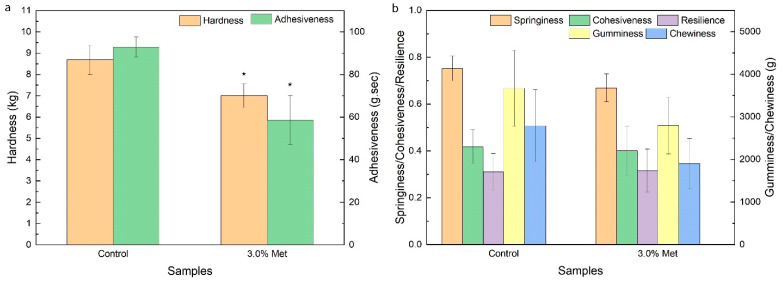
Textural profile analysis of BC membranes. (**a**) Hardness and adhesiveness of the BC membranes; (**b**) Springiness, cohesiveness, gumminess, chewiness, and resilience of the BC membranes. Control: the BC membrane obtained in the FCW medium; 3.0% Met: the BC membrane obtained in the FCW medium with the addition of 3.0% methionine. Significant differences of the samples to the control group were confirmed at * *p* < 0.05 (n = 3).

TPA was performed to investigate the texture characteristics of BC membranes obtained with and without the addition of 3.0% methionine. As shown in Figure 6, the hardness and adhesiveness of BC membranes decreased by 19.3% and 37.0% under the addition of 3.0% methionine, respectively. Hardness reflects the energy required to deform to a certain degree, but adhesiveness represents the capacity to adhere to other surfaces [42]. The decreased hardness of BC under the addition of 3.0% methionine may improve the shape formation ability of BC [42], but the decrease in adhesiveness might limit its application in the food additives industry [43,44]. The texture properties might be related to the chemical characteristics and microstructural features of membranes, such as porosity, network crosslinking, and interconnections between fibril layers [45]. Although the crystallinity degree of the BC membrane increased and the signal absorption intensity changed, no denser structure formed under the addition of 3.0% methionine. The thinner, longer, and looser fibrils of BC might reduce its deformation resistance under the addition of 3.0% methionine. The altered hardness and adhesiveness of BC membrane did not affect other textural properties under the addition of 3.0% methionine. The two groups of BC membranes displayed no obvious changes in springiness, cohesiveness, gumminess, chewiness, and resilience, which suggested that the addition of 3.0% methionine may not affect the taste of nata de coco or the consumers’ preference to nata de coco. These results were consistent to those of Jacek et al., in which the texture characteristics of BC membranes yielded by the same strain variedly changed in different media [45].

## 4. Conclusions

The results of this study suggest that the coconut water pre-fermented by different coconut-sourced yeast strains had varied effects on the production of BC from *K. nataicola* Q2. Compared with that in the FCW medium, the production of BC increased by 165% in the SC7-PCW medium, which was slightly higher than that obtained in the NPCW medium. Therefore, using coconut water pre-fermented by SC7 is expected to be a potential alternative strategy to achieve safety, stable, and high-yield production of nata de coco. Both natural pre-fermentation and SC7 pre-fermentation altered the concentration of amino acids of FCW. The addition of selected amino acids at different concentrations had varied effects on the production of BC. The yield of BC was the highest under the addition of 3.0% methionine. Specifically, the addition of 3.0% methionine allowed the production of a BC membrane with larger loops of looser aggregated microfibers, higher crystallinity but lower thermal stability, hardness, and adhesiveness in the test condition. The variety and concentration of amino acids could be adjusted according to the specific application requirements of BC in future.

The price of BC largely depends on the form (such as dry film and gel) of BC and its application area. Compared with that in the synthetic medium HS medium, the yield of BC obviously increased in the media of FCW, NPCW, and SC7-PCW using the strain *K. nataicola* Q2 with decreased production cost. The profit of using FCW, NPCW, and SC7-PCW media is thus higher than that of using HS medium, despite that BC is sold in the form of dry film or gel in various applications. The addition of methionine increases the raw material cost of BC production. It is economical to add methionine in FCW medium when BC is sold as dry films with a higher selling price; but when BC is sold as wet films with a lower selling price, the method of adding methionine to coconut water is not as cost-effective as that without adding methionine. Even at a very low selling price, the profit of using the FCW medium with the addition of 3.0% methionine is lower than that of using the HS medium. Therefore, it is necessary to consider the production cost, efficiency, product positioning, and market demand to determine the production process of BC. This research provides a guidance for the increased production and modification of BC and offers a new perspective for the study of the metabolic regulation of BC.

## Data Availability

Data is contained within the article or supplementary material.

## Supplementary Materials

The following supporting information can be downloaded at: https://www.mdpi.com/article/10.3390/foods11223627/s1. Figure S1. Phylogenetic trees. (a) Phylogenetic tree based on the 16S rDNA sequence of the BC-yielding strain Q2; (b) Phylogenetic trees based on the ITS sequences of yeast strains 4, 34, 7, 20, and 9 and the 26S rDNA sequences of strains 81, 71, 74, 84, 134, and 117. Sequencing results were analyzed using NCBI Blast (https://blast.ncbi.nlm.nih.gov/Blast.cgi). Multiple sequences alignment was conducted by a MAGA11 software using the p-distance method. Phylogenetic trees were constructed using a Neighbor-joining method. Figure S2. Effect of the addition of ethanol, acetic acid, and pyruvic acid on the production of BC. FCW: fresh coconut water medium; Ethanol: FCW medium containing 1.0% ethanol; Acetic acid: FCW medium containing 1.0% acetic acid; Pyruvic acid: FCW medium containing 1.0% pyruvic acid. Significant differences of the samples to the control group FCW were confirmed at * *p* < 0.05 (n = 3). Figure S3. XRD diffractograms (a) and thermogravimetry curves (b) of BC membranes. Control: the BC membrane obtained in the FCW medium; 3.0% Met: the BC membrane obtained in the FCW medium with the addition of 3.0% methionine.
