# Supplementary material for: Production of Bacterial Cellulose in the Medium with Yeasts Pre-Fermented Coconut Water or with Addition of Selected Amino Acids
Source: Foods. 2022 Nov 14;11(22):3627. doi: 10.3390/foods11223627 (PMC9689973; doi:10.3390/foods11223627)
Supplement: Supplementary file 1 [file foods-11-03627-s001.zip › foods-1942067-supplementary.pdf]

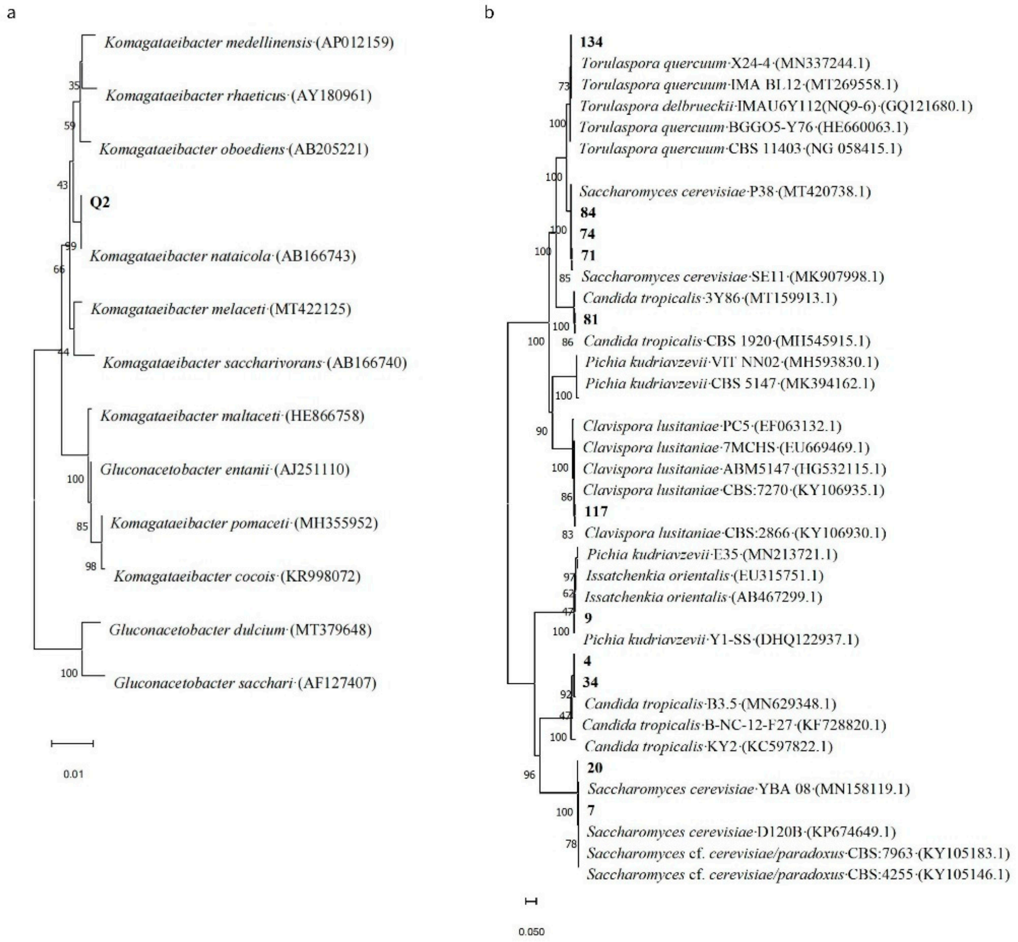

Figure S1 Phylogenetic trees. (a) Phylogenetic tree based on the 16S rDNA sequence of the BC-yielding strain Q2; (b) Phylogenetic trees based on the ITS sequences of yeast strains 4, 34, 7, 20, and 9 and the 26S rDNA sequences of strains 81, 71, 74, 84, 134, and 117. Sequencing results were analyzed using NCBI Blast (https://blast.ncbi.nlm.nih.gov/Blast.cgi). Multiple sequences alignment was conducted by a MAGA11 software using the p-distance method. Phylogenetic trees were constructed using a Neighbor-joining method.

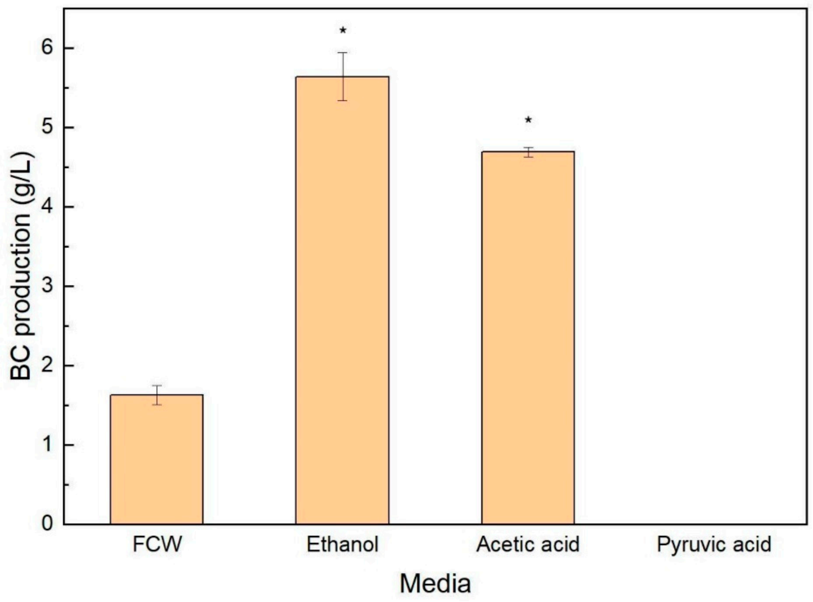

Figure S2 Effect of addition of ethanol, acetic acid, and pyruvic acid on the production of BC. FCW: fresh coconut water medium; Ethanol: FCW medium containing 1.0% ethanol; Acetic acid: FCW medium containing 1.0% acetic acid; Pyruvic acid: FCW medium containing 1.0% pyruvic acid. Significant differences of the samples to the control group FCW were confirmed at * $p < 0.05$ (n=3).

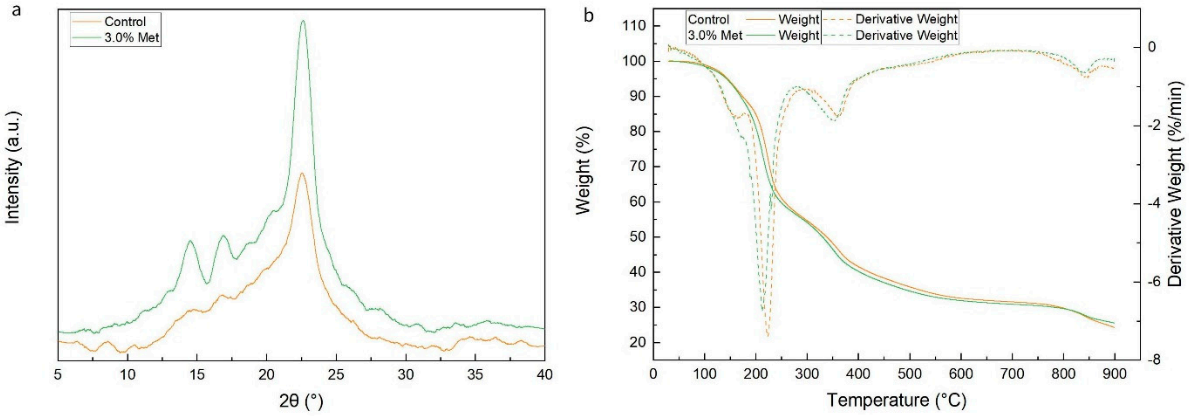

Figure S3 XRD diffractograms (a) and thermogravimetry curves (b) of BC membranes. Control: the BC membrane obtained in the FCW medium; 3.0% Met: the BC membrane obtained in the FCW medium with the addition of 3.0% methionine.
